# A phenocopy signature of *TP53* loss predicts response to chemotherapy

**DOI:** 10.1038/s41698-024-00722-7

**Published:** 2024-10-02

**Authors:** Hamza Bakhtiar, Marina N. Sharifi, Kyle T. Helzer, Yue Shi, Matthew L. Bootsma, Tianfu A. Shang, Matthew R. Chrostek, Tracy J. Berg, S. Carson Callahan, Viridiana Carreno, Grace C. Blitzer, Malinda T. West, Ruth M. O’Regan, Kari B. Wisinski, Martin Sjöström, Shuang G. Zhao

**Affiliations:** 1 Department of Human Oncology, University of Wisconsin, Madison, WI USA; 2 Department of Medicine, Division of Hematology, Oncology, and Palliative Care, University of Wisconsin, Madison, WI USA; 3 Carbone Cancer Center, University of Wisconsin, Madison, WI USA; 4 Department of Medicine, University of Rochester, Rochester, NY USA; 5 Department of Radiation Oncology, University of California San Francisco, San Francisco, CA 94143 USA; 6 William S. Middleton Memorial Veterans Hospital, Madison, WI USA

**Keywords:** Molecular medicine, Computational biology and bioinformatics

## Abstract

In preclinical studies, p53 loss of function impacts chemotherapy response, but this has not been consistently validated clinically. We trained a *TP53*-loss phenocopy gene expression signature from pan-cancer clinical samples in the TCGA. In vitro, the *TP53*-loss phenocopy signature predicted chemotherapy response across cancer types. In a clinical dataset of 3003 breast cancer samples treated with neoadjuvant chemotherapy, the *TP53*-loss phenocopy samples were 56% more likely to have a pathologic complete response (pCR), with a significant association between *TP53*-loss phenocopy and pCR in both ER positive and ER negative tumors. In an independent clinical validation in the I-SPY2 trial (*N* = 987), we confirmed the association with neoadjuvant chemotherapy pCR and found higher rates of chemoimmunotherapy response in *TP53*-loss phenocopy tumors compared to non-*TP53*-loss phenocopy tumors (64% vs. 28%). The *TP53-*loss phenocopy signature predicts chemotherapy response across cancer types in vitro, and in a proof-of-concept clinical validation is associated with neoadjuvant chemotherapy response across multiple clinical breast cancer cohorts.

## Introduction

p53 is a transcription factor that drives a broad range of anti-proliferative programs in response to diverse intrinsic and extrinsic cellular stressors^1^. Consequently, it plays a key tumor suppressor role across tissue types, and is the most commonly altered gene in cancer, with somatic mutations observed in almost all cancer types at rates ranging from 5% to as high as 90%^2^. The spectrum of *TP53* somatic mutations is incredibly diverse, with over 2000 different variants described^3^. About 80% of *TP53* mutations occur in the DNA binding domain (DBD), including 6 hotspots that comprise 25% of all *TP53* mutations and almost 90% of these DBD mutations are missense mutation that alter DNA binding^2^. Conversely, about 60% of mutations outside the DBD are nonsense or truncating mutations^2^. While the majority of *TP53* mutations result in loss of the normal function of the protein, some mutations also appear to have dominant-negative and/or gain-of-function oncogenic effects^4^. In addition, almost all *TP53* mutated tumors exhibit alteration or loss of second allele function through biallelic mutation, loss-of-heterozygosity (LOH), or chromosomal deletion^5^. Given this complexity of the *TP53* alteration landscape and despite the remarkable depth of pre-clinical data characterizing the impact of diverse *TP53* mutations on protein function^6–8^ it is not possible to interpret the anticipated function of *TP53* mutations in patient tumors based on DNA mutation status alone, limiting the development of predictive or prognostic biomarkers and novel targeted therapy approaches based on this frequently mutated and crucial tumor suppressor.

In preclinical studies, p53 protein loss of function has a profound impact on cellular responses to DNA-damaging agents including cytotoxic chemotherapies and radiation^9–11^, leading to the hypothesis that *TP53* alterations may serve as a biomarker for sensitivity to cytotoxic chemotherapies. Breast cancer has a high frequency of *TP53* DNA alterations, which are found in up to 30-40% of cases^12,13^, and are associated with poor prognosis^14–17^, though this is modulated both by *TP53* alteration type^17,18^ and breast cancer subtype^19–21^. There has consequently been extensive investigation of p53 status and neoadjuvant chemotherapy response in early-stage breast cancer, with conflicting results. Some smaller cohort studies have found an association between *TP53* DNA alterations and chemotherapy response^22–24^, while others have found the inverse^17,25,26^. Likewise, while some smaller case series found an association between low p53 protein expression and chemotherapy response^27^, others associated p53 overexpression with chemotherapy response^22,28^, and several found no association between p53 protein and response^23,29^. In many cases, these studies may have been confounded by heterogeneity in the spectrum of detected *TP53* alterations as well as heterogeneity in breast cancer subtype, which impacts both frequency and type of *TP53* alteration and neoadjuvant therapy response rates. However, the association between *TP53* DNA alteration status and breast cancer neoadjuvant chemotherapy response has also been evaluated in in two large clinical trial cohorts: EORTC 10944/BIG 1-00^30^, a phase III trial comparing anthracycline versus anthracycline/taxane neoadjuvant therapy across breast cancer subtypes and the GeparSixto phase II trial^31^ evaluating anthracycline/taxane/platinum-based neoadjuvant therapy in triple-negative and HER2-positive breast cancer. Neither of these trials found a significant association between *TP53* DNA alteration status and neoadjuvant chemotherapy response.

DNA alterations can create up and down-stream changes in gene expression throughout a pathway. However, not all DNA alterations are functional, and conversely, there can be other genomic, epigenomic, or transcriptional changes that produce a similar gene expression pattern to a pathogenic DNA alternation (i.e. a phenocopy). Gene expression-based signatures may provide complementary information to DNA alteration status alone in predicting treatment response. For example, an RNA-based signature for p53 pathway activity has been shown to be more strongly associated with survival than DNA *TP53* status in a large breast cancer cohort^32^. We have similarly created RNA-based phenocopy signatures for multiple DNA alterations (besides *TP53*) that serve as indications for targeted therapy and demonstrated that the phenocopy signatures added to the DNA alterations in predicting treatment response to those same targeted agents^33^. Herein, we used this same strategy to develop a new RNA-based phenocopy signature for *TP53*-loss as a predictor for cytotoxic chemotherapy response.

## Results

### *TP53*-loss phenocopies are enriched for DNA alterations

**Fig. 1:**
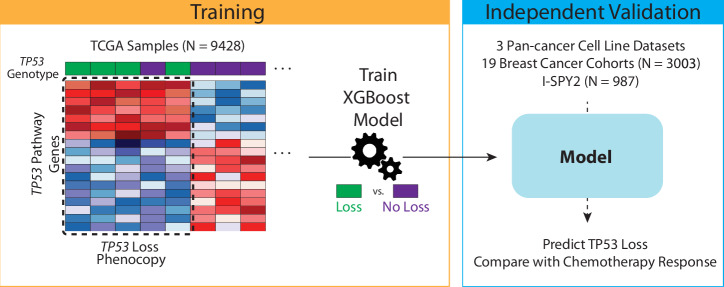
Schematic of *TP53*-loss phenocopy signature model development. In the TCGA training cohort, *TP53*-loss genomic status (purple – genomic T*P53* loss sample, green – no genomic *TP53* loss sample) and expression of p53-pathway relevant genes are utilized to generate a *TP53*-loss phenocopy transcriptional signature using an XGBoost machine learning model (Training, left). To validate this model, the *TP53*-loss phenocopy signature is then used to predict phenocopy status from expression data in cell line databases (GDSC, CCLE, DepMap) and predicted phenocopy status (phenocopy versus not) is compared to genomic *TP53*-loss across cancer cell lines for predicting chemotherapy response (Independent Validation, left). Finally, phenocopy status is predicted from expression data across the breast cancer clinical cohorts and association with neoadjuvant chemotherapy response is evaluated (Independent Validation, left).

Using our previously published machine learning approach^33^ we trained and locked a pan-cancer *TP53-*loss phenocopy model in 9428 clinical samples from The Cancer Genome Atlas (TCGA) as described in the methods (Fig. 1). We focused on the subset of tumors with dual *TP53* DNA alterations as these most likely represent biallelic loss-of-function alterations, thus avoiding potential uncertainty about monoallelic *TP53* alterations.

**Fig. 2:**
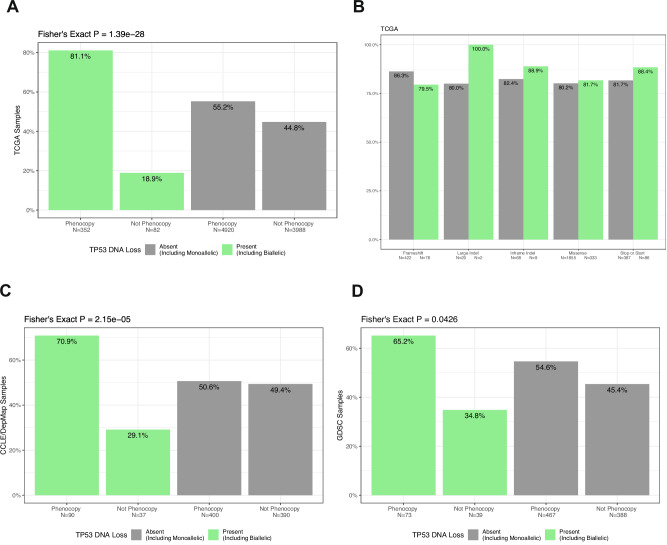
Association between DNA mutation status and *TP53*-loss phenocopy prediction in the pan-cancer TCGA cohort. **A** In the TCGA pan-cancer cohort (*n* = 9428), significantly more dual *TP53* DNA loss (assumed to be bi-allelic) samples were predicted to be RNA *TP53-*loss phenocopies, but the rate was also high in the non-*TP53-loss* group (81% vs 55%) as many of these samples also harbor single mono-allelic *TP53* alterations. **B** For each type of *TP53* mutation, the rates of predicted phenocopy status were similar in the dual (bi-allelic) vs. single (mono-allelic) *TP53* DNA loss group. Similar to TCGA, in the (**C**) CCLE and the (**D**) GDSC cell line cohorts, significantly more *TP53* DNA loss samples were predicted to be RNA *TP53* loss phenocopies.

We then examined the association between the *TP53-*loss phenocopy signature and DNA alterations in the TCGA cohort. As in the training, *TP53* DNA loss was defined as only including dual, and therefore presumed bi-allelic, *TP53* alterations to be as specific as possible for tumor suppressor loss and exclude any mono-allelic oncogenic *TP53* alterations. As expected, the vast majority (81%; Fig. 2A) of *TP53-*loss genotype samples in the TCGA cohort (*N* = 9428) were predicted to be an RNA *TP53-*loss phenocopy. However, while some of the mono-allelic *TP53* altered samples may be oncogenic or non-pathogenic, many actually confer a tumor suppressor function. This is reflected in the relatively high rate (55%; Fig. 2A) of predicted *TP53* phenocopies in the samples without a bi-allelic *TP53-*loss genotype. When we further examined the phenocopy rates across different *TP53* mutation types in the samples with a bi-allelic vs. mono-allelic *TP53* DNA alteration, we found that the rates of predicted phenocopy status were fairly similar (Fig. 2B), consistent with this hypothesis. The majority of *TP53-*loss genotype samples in the CCLE and GDSC cell line datasets were also predicted to be an RNA *TP53-*loss phenocopy similar to TCGA (Fig. 2C, D). We also investigated a gene set comparing p53 mutated versus non-mutated cell lines in the NCI-60 collection^34^ for GSVA single sample gene set analysis^35^ in the TCGA samples: P53_DN.V1_UP (UPregulated in *TP53* mutated) and P53_DN.V1_DN (DowNregulated in *TP53* mutated). *TP53* phenocopy samples with or without *TP53* DNA loss have similar *TP53*-mutated-UP and *TP53*-mutated-DN signature scores, consistent with similar levels of *TP53* dysfunction in both DNA loss and non-DNA loss phenocopy groups. Likewise, non-*TP53* phenocopy samples with or without DNA loss have similar *TP53*-mutated-UP and *TP53*-mutated-DN signature scores. However, in samples without *TP53* DNA loss, the *TP53* phenocopy samples have higher *TP53*-mutated-UP and lower *TP53*-mutated-DN signature scores, again consistent with differing level of p53 dysfunction between the phenocopy and non-phenocopy samples (Supplementary Fig. 1).

### *TP53*-loss phenocopy status predicts response to chemotherapy across three pan-cancer in vitro studies

**Fig. 3:**
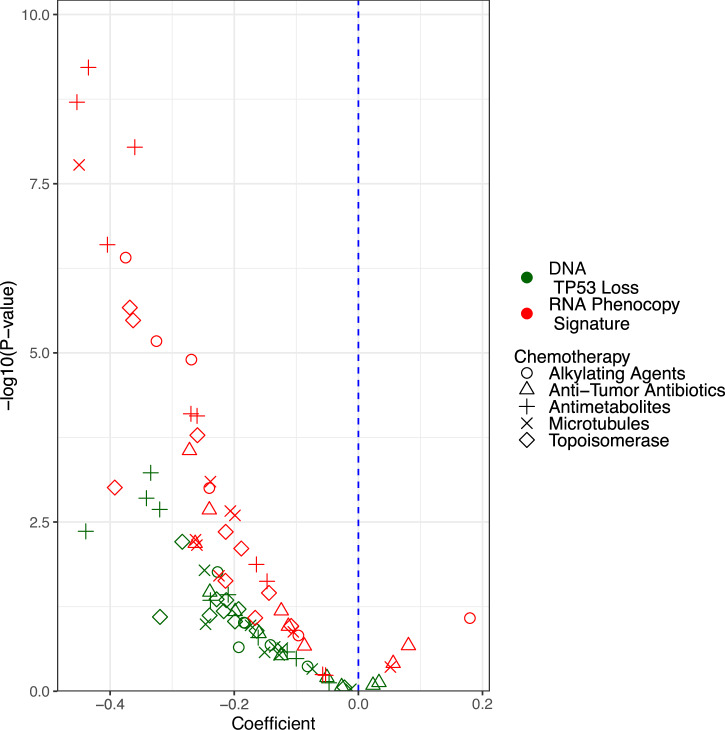
*TP53*-loss phenocopy signature predicts chemotherapy response in vitro pan-cancer. Linear models for cytotoxic chemotherapy response were used to assess how much the RNA-based *TP53-*loss phenocopy signature added to DNA-based genomic *TP53* loss in the cell line datasets. Each model of a single chemotherapy is represented by two points, one for each independent variable (genomic *TP53* loss in green, *TP53*-loss phenocopy signature in red). The x-axis represents the linear coefficient, and the y-axis is the associated -Log10(p-value) of each independent variable in the linear model. Negative coefficients represent expected estimates, where the genomic or phenocopy *TP53-*loss status is associated with increased sensitivity to each chemotherapy. Data points in the upper-left quadrant therefore represent drugs for which the phenocopy signature most significantly contributed to predicting chemotherapy sensitivity.

We next sought to determine if our *TP53-*loss phenocopy signature was associated with sensitivity to cytotoxic chemotherapies. We utilized three pan-cancer cell line datasets (GDSC: *N* = 950; CCLE and DepMap which share NGS data: *N* = 917) that contain response data for many cytotoxic chemotherapies across large panels of cancer cell lines annotated with both genomic and gene expression profiling. To investigate whether *TP53-*loss phenocopy status predicts chemotherapy response, we created multi-variable linear models with both the RNA phenocopy signatures and DNA *TP53-*loss status (as defined above to include only bi-allelic alterations) predicting response for each chemotherapy. We found that across most cytotoxic chemotherapies and classes in all three datasets, the phenocopy signatures predicted chemotherapy response independently and to a greater degree than DNA *TP53-*loss status alone (Fig. 3, Supplementary Fig. 2). The in-vitro validation of this signature to predict chemotherapy response beyond DNA alterations alone supports further investigation in clinical datasets.

### *TP53*-loss phenocopy status is associated with neoadjuvant chemotherapy response in breast cancer clinical samples

**Fig. 4:**
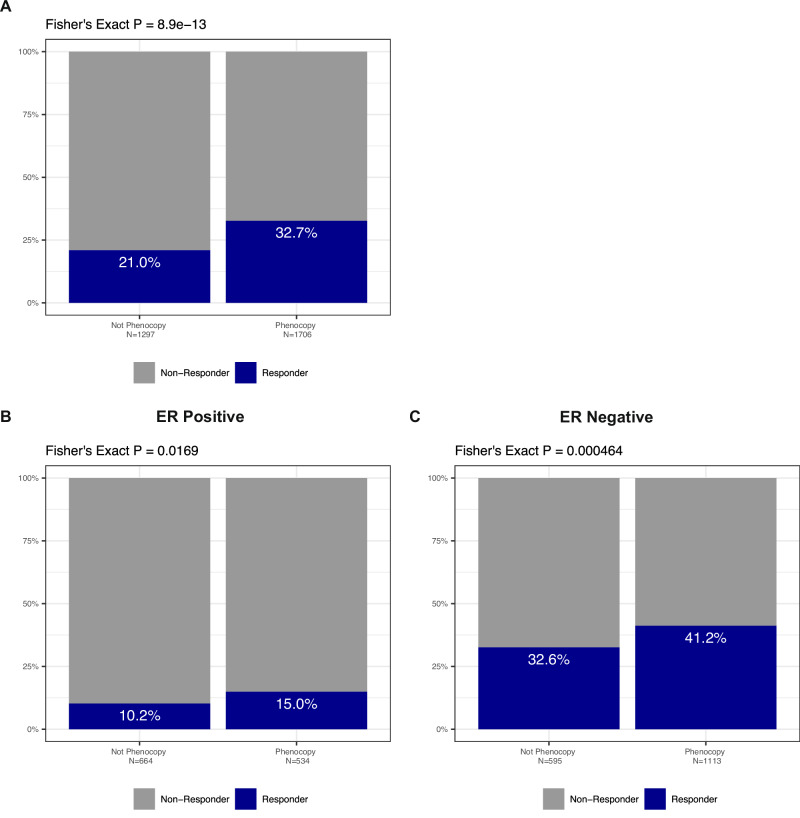
*TP53*-loss phenocopy signature is associated with neoadjuvant chemotherapy response in breast cancer. **A** *TP53*-loss phenocopy status is significantly associated with pathologic-complete response to neoadjuvant chemotherapy in a combined cohort of 3,003 early-stage breast cancers. This association is seen in both ER positive (**B**) and ER-negative (**C**) breast cancers.

For an initial clinical validation of our *TP53-*loss phenocopy signature, we sought to identify a clinical context with a relatively uniform approach and standardized definition of chemotherapy response. One such clinical context is pathologic response to neoadjuvant chemotherapy in early-stage breast cancer^36^, particularly the HER2-positive and triple-negative subtypes where pathologic response to neoadjuvant chemotherapy is strongly associated with prognosis and recurrence risk^37^. Importantly, recurrent *TP53* DNA alterations are relatively common in breast cancer, and the role of genomic *TP53* loss has been extensively studied in this setting and has not reliably predicted chemotherapy response^30,31^. As such, we set out to investigate the ability of our *TP53-*loss phenocopy signature to predict pathologic complete response to breast cancer neoadjuvant chemotherapy. To address this question, we examined 19 breast cancer datasets comprising a total of 3003 samples (Supplementary Table 1) with pre-treatment gene expression profiling and pathologic response annotation. All patients were treated with neoadjuvant cytotoxic chemotherapy following tissue profiling, and pathologic response assessment was performed at the time of surgery. In total, we found that 33% of the phenocopy samples had a pathologic complete response (pCR), compared to 21% of the non-phenocopy samples (Fisher’s Exact *P* < 0.0001, Fig. 4A). *TP53-*loss phenocopy status predicted pCR independently from pathologic grade and T/N-stage in two of the larger, more recent cohorts treated with modern anthracycline/taxane chemotherapy (Supplementary Fig. 3, Supplementary Table 2).

ER-positive breast cancers have much lower rates of *TP53* DNA alterations than ER-negative (HER2 positive and triple negative) tumors^13^. Additionally, ER-positive tumors have much lower rates of pCR after neoadjuvant chemotherapy than ER-negative tumors reflecting relative chemoresistance of the ER-positive subtype^38^. We found that RNA phenocopy signature rates differed by ER status, with 44.6% of ER-positive samples compared to 65.2% of the ER-negative samples predicted as *TP53* phenocopies (Fig. 4B, C). Intriguingly, while overall rates of pCR were lower for ER-positive cancers as expected (12.4% in ER-positive versus 38.2% in ER-negative) there was still a significant association between phenocopy status and likelihood of pCR, seen in 15.0% of the phenocopy samples compared to 10.2% of the non-phenocopy samples in ER-positive disease (Fisher’s Exact *P* = 0.0169, Fig. 4B). Likewise, for ER-negative disease, 41.2% of the phenocopy samples had a pCR, compared to 32.6% of the non-phenocopy samples (Fisher’s Exact *P* = 0.0005, Fig. 4C). Taken together, this demonstrates that our RNA *TP53-*loss phenocopy signature is associated with chemotherapy response in this clinical context, providing proof of principle validation of our in-vitro results.

### *TP53*-loss phenocopy status predicts residual disease burden in triple-negative breast cancer in the BrighTNess Phase III clinical trial

**Fig. 5:**
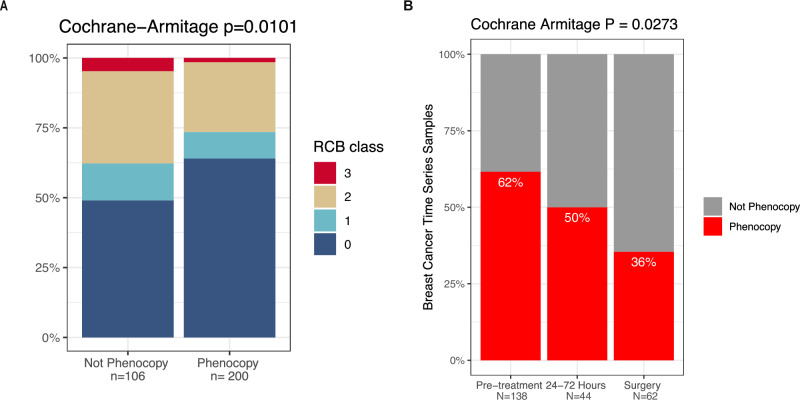
*TP53*-loss phenocopy signature is associated with residual cancer burden in the BrighTNess trial and decreases during neoadjuvant chemotherapy in the I-SPY1 trial. **A** *TP53*-loss phenocopy status is significantly associated with RCB class after carboplatin-containing neoadjuvant therapy in the phase III BrighTNess clinical trial in triple negative breast cancer. **B** In serial tissue samples before, during and after neoadjuvant therapy in the I-SPY1 clinical trial, *TP53*-loss phenocopy proportion decreases over the course of neoadjuvant therapy.

Despite recent treatment advances, early-stage triple-negative breast cancers continue to have a worse prognosis than other breast cancer subtypes. In patients with residual disease after neoadjuvant chemotherapy, the pathologic residual cancer burden (RCB) method to quantify extent of residual disease has been shown to have significant prognostic value in stratifying recurrence risk^39^. This method classifies residual disease status as RCB-0 (pCR), RCB-I, RCB-II, and RCB-III based on standardized pathologic parameters, with a higher class indicative of greater extent of residual disease and associated with higher recurrence risk. We leveraged gene expression samples from the phase III BrighTNess clinical trial of neoadjuvant anthracycline/taxane therapy +/- platinum and PARP inhibitor veliparib in triple-negative breast cancer^40^ for which RCB class annotation was available to evaluate the association between pre-treatment phenocopy status and RCB class. Due to a significant difference in the rate of pathologic complete response between the non-carboplatin and carboplatin arms of the trial seen in primary analysis^41^, we specifically focused this analysis on the carboplatin-treated samples. We observed that there was a significantly lower burden of residual disease across RCB categories in the phenocopy group compared to the not phenocopy group (Cochran-Armitage *P* = 0.0101, Fig. 5A), consistent with a continuous association of *TP53-*loss phenocopy status with magnitude of chemotherapy response, at least in triple negative breast cancer.

### *TP53*-loss phenocopy proportion decreases during course of chemotherapy in the I-SPY1 clinical trial

In addition, one of the clinical breast cancer cohorts collected time-series data before, during, and after treatment with neo-adjuvant chemotherapy from the I-SPY1 trial^42^. We examined the proportion of samples predicted as phenocopies at each time point. Pre-treatment, 62% of samples were predicted as phenocopies. However, this decreased to 50% by 24–72 h on-treatment, and further decreased to 36% by the end of chemotherapy on the surgical specimen (Cochran-Armitage *P* = 0.0273, Fig. 5B). If our *TP53-*loss phenocopy indeed predicts chemo-sensitivity as our in-vitro and clinical data suggest, we would expect that chemotherapy would cause a reduction in phenocopy tumor cells as the most sensitive cells are killed, matching our observation in this dataset.

### *TP53*-loss phenocopy status is associated with response to neoadjuvant chemo-immunotherapy in triple negative breast cancer in the I-SPY2 clinical trial

**Fig. 6:**
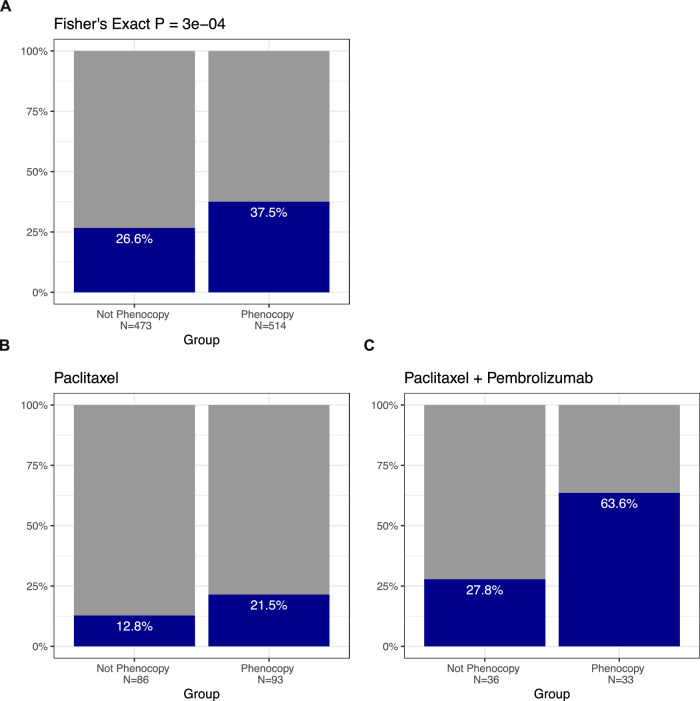
*TP53*-loss phenocopy signature is associated with neoadjuvant chemoimmunotherapy response in the I-SPY2 trial. **A** Validation study demonstrating that *TP53*-loss phenocopy status is significantly associated with pathologic complete response to anthracycline/taxane-based chemotherapy across all arms of the I-SPY2 trial. Compared to standard anthracycline/taxane chemotherapy (**B**), phenocopy status is associated with a particularly high rate of pathologic complete response to chemoimmunotherapy (**C**).

Given the inclusion of some older studies with now-obsolete chemotherapy regimens in our initial validation cohort studies, we performed a second independent clinical validation of our *TP53-*loss phenocopy signature leveraging the I-SPY2 990 Data Resource^38^. This dataset comprises gene expression and clinical response data from 987 patients enrolled in the I-SPY2 adaptive neoadjuvant trial platform with an anthracycline/taxane-based neoadjuvant chemotherapy backbone, which represents a standardized and modern cytotoxic chemotherapy backbone regimen. *TP53-*loss phenocopy status was also significantly associated with pathologic complete response overall in I-SPY2, with a 38% pCR rate in the phenocopy samples versus 27% of non-phenocopy samples (Fisher’s Exact *P* = 0.0003, Fig. 6A). Importantly, one arm of I-SPY2 was treated with chemoimmunotherapy (with pembrolizumab) which has subsequently become standard of care for most triple-negative breast cancers after the demonstration of both increased pCR rates and improved event free survival with the addition of pembrolizumab to chemotherapy in the randomized phase III Keynote 522 trial^43^. Compared to the chemotherapy only control arm (Fig. 6B), the pCR rate was overall higher in the I-SPY2 chemoimmunotherapy arm (Fig. 6C) concordant with the subsequent findings from the Keynote 522 trial^44^. However, while the pCR rates and delta between phenocopy and non-phenocopy groups were similar in the chemotherapy only arm (Fig. 6B) to what we observed in our prior aggregate chemotherapy response dataset (Fig. 4), the pCR rate in the chemoimmunotherapy phenocopy samples as well as the delta between phenocopy and non-phenocopy samples was disproportionately higher at 64% in the phenocopy samples versus 28% in the non-phenocopy samples (Fig. 6C; Fisher’s Exact = 0.00376). While a preliminary finding in a small cohort, this raises the intriguing possibility that *TP53* functional status may play a role not only in response not only to cytotoxic chemotherapy but also to chemo-immunotherapy combination regimens.

## Discussion

In our manuscript, we describe a pan-cancer phenocopy signature for *TP53-*loss trained in a cohort of 9,428 clinical samples from the Cancer Genome Atlas (TCGA). We then validate that the *TP53-*loss phenocopy signature predicts for sensitivity to chemotherapy beyond DNA *TP53* mutation status and across tumor types in three large cell line databases. For an initial clinical proof of concept validation, we aggregated 3,003 breast cancer biopsy samples across 19 cohorts and identified a significant association between our *TP53-*loss phenocopy signature and pathologic complete response and extent of residual disease after neoadjuvant chemotherapy in early-stage breast cancer. Finally, we performed further independent clinical validation in the I-SPY2 breast cancer neoadjuvant chemotherapy adaptive trial (*N* = 987) confirming the significant association between *TP53-*loss phenocopy status and pathologic complete response.

In contrast to prior studies focused on DNA alteration status^30,31^, we have been able to identify a robust association between our gene-expression-based signature and chemotherapy response in early-stage breast cancer by focusing on a transcriptional profile most consistent with *TP53* loss of function. This is concordant with a biomarker analysis of the I-SPY1 trial, where an RNA-based p53 mutation signature was associated with pCR as well as subsequent risk of disease recurrence^45^. Importantly, we also identified a significant association between our *TP53-*loss phenocopy status and residual disease burden after standard neoadjuvant chemotherapy, suggesting that our signature is continuously associated with degree of chemosensitivity.

Intriguingly, we saw a particularly strong association between *TP53-*loss phenocopy status and chemoimmunotherapy response in triple-negative breast cancer. The impact of tumor cell-intrinsic *TP53* alterations on anti-tumor immune responses is complex^46^. However, *TP53* mutations have been associated with increased response to chemoimmunotherapy in non-small cell lung cancer^47^ and mutated *TP53* has been shown to induce anti-tumor T cell responses as a neoantigen in ovarian cancer^48,49^. It is possible that similar mechanisms play a role in chemoimmunotherapy response in triple-negative breast cancer which has the highest rate of *TP53* mutations across breast cancer subtypes^19^.

Chemosensitivity is a complex phenotype that can be mediated by multiple factors beyond p53 function, including upregulation of drug efflux pumps, intracellular drug metabolism pathways, increased DNA repair pathway activity and reduced apoptosis^50^ and changes in the tumor microenvironment and tumor-immune interface^51^. While p53 functional status is a component, this complexity is reflected in the modest effect size that we observed in our clinical validation cohorts. However, the *TP53-*loss phenocopy signature is clearly associated with neoadjuvant chemotherapy response in breast cancer across multiple clinical cohorts comprising nearly 4000 patients, including patients treated with chemoimmunotherapy. Additionally, as our *TP53-*loss phenocopy signature was trained on clinical samples across cancer types and validated in pan-cancer in vitro datasets for chemotherapy response, while our initial clinical validation focused on early-stage breast cancer, there is the potential for broader applicability across tumor types, though this would require additional clinical validation.

## Methods

### *TP53*-loss phenocopy RNA signature development

We sought to mirror our previous phenocopy signature development approach as much as possible. First, we wanted to focus on *TP53* alterations leading to the tumor suppressor loss. The presence of two DNA alterations in *TP53* most likely represents bi-allelic loss of function. Therefore, we defined *TP53* DNA loss as either two *TP53* coding mutations, or a single coding mutation and CN loss. While many single *TP53* alteration samples may have a tumor suppressor phenotype through LOH or a dominant negative phenotype, others may have an oncogenic phenotype. We prioritized specificity in training the model, and thus using only the most confident examples of *TP53-*loss-of-function was more important than sensitively identifying all examples. In addition, MDM2 is an E3 ubiquitin ligase that plays a key role in regulating p53 through proteasomal degradation, which can also lead to loss of wild-type p53 activity and mimic the tumor-promoting phenotype of *TP53* genomic loss of function^52^. Thus, we also included alterations in MDM2 as previously described^33^. This set of alterations is referred to as “*TP53* loss” throughout the manuscript. Likewise, we utilized the public calls for mutation and CN in TCGA, GDSC, and CCLE/DepMap without modification^33^. For TCGA, a GISTIC threshold of ≤−1 was used for CN loss. For GDSC, a GISTIC threshold of <−1 was used for CN loss. In CCLE, CN loss was defined as a Log_2_ CN < −1. We again utilized the Reactome database to identify a list of genes associated with *TP53* status with which to train our RNA phenocopy model. We selected the “*TP53* Regulates Transcription of Cell Cycle Genes” pathway as transcription is what we are measuring, and the cell cycle pathway is the target of cytotoxic chemotherapies. We then trained a gradient-boosted tree (XGBoost) model using TCGA pan-cancer samples, similar to our prior efforts^33^ (Supplementary Fig. 4).

### Gene expression normalization and batch correction

Given the diversity of gene expression platforms across in-vitro and clinical validation datasets, we performed batch correction and normalization using the same method as previously described^33,53^. In brief, every sample was rank normalized with a ‘dense’ method for ties. For GDSC and CCLE/DepMap we then performed batch correction using the R SVA package (COMBAT) across all cancer types with TCGA as the reference (as these datasets have a large number of cancer types). For the breast cancer datasets, we performed the COMBAT batch correction step only against ‘breast invasive carcinoma’ (BRCA) samples within TCGA. There was no missing data in GDSC or CCLE/DepMap. Some of the breast cancer clinical studies had gene expression data missing for a small number of genes in certain samples. In these cases, we imputed the average gene expression for that gene across all the other samples in that cohort.

### Independent validation in vitro

We next validated our model in the GDSC, CCLE, and DepMap cell line databases. GDSC had both DNA/RNA data, but CCLE and DepMap shared DNA/RNA data with independent drug response assessments. We identified all cytotoxic chemotherapies assessed in these cohorts. The *TP53-*loss phenocopy signature was then applied without modification to the GDSC and CCLE/DepMap, with normalization/batch correction as above. Although there is some overlap between chemotherapeutic agents used in each cohort, we chose to analyze each cohort independently as each study was done separately in a manner as previously described^33^. Briefly, to assess if the phenocopy signature predictions were associated with chemotherapy response, we created a linear model for each drug with the dependent variable as a measurement of drug sensitivity. This was the Z-score of the IC50 in GDSC, -ActArea in CCLE, and -AUC in DepMap, such that a lower score was associated with increased drug sensitivity^33^. The independent variables in the linear model were then the *TP53* RNA phenocopy score, and DNA *TP53* loss as defined above.

### Breast cancer clinical validation

The *TP53-*loss phenocopy signature was then applied without modification to pre-treatment samples from each breast cancer study, with normalization/batch correction as above. ER-positive versus ER-negative status as well as annotation for pathologic response (pCR versus no pCR) were extracted from GEO metadata for each cohort (Supplementary Table 1). We then compared the responder (pCR)/non-responder (no pCR) proportions in the phenocopy vs. not phenocopy groups using Fisher’s exact test. The GSE164458 cohort from the phase III BrighTNess trial^40^ in triple negative breast cancer was also annotated for residual cancer burden (RCB) status in patients with no pCR, and proportion of each RCB class in phenocopy versus not phenocopy groups was compared using a Cochrane-Armitage test. Furthermore, the GSE32603 cohort from the I-SPY1 trial^42^ contained pre-treatment, on-treatment (24–72 h), and post-treatment (surgery) samples, allowing us to assess how the phenocopy predictions changed longitudinally, with the significance of the change in the proportion of pCR/no pCR samples at each timepoint compared using a Cochrane-Armitage test. The GSE194040 cohort from the I-SPY2 clinical trial^38^ was used as an independent clinical validation set, and to evaluate the association between the *TP53-*loss phenocopy signature and chemoimmunotherapy response.

## Supplementary information

Supplemental Data

## Data Availability

As in our previous phenocopy publication^33^, we utilized TCGA as our training dataset and GDSC, CCLE, and DepMap as our in vitro validation datasets. Processed DNA and RNA sequencing data from TCGA were downloaded using the UCSC Xena browser (xena.ucsc.edu). Processed DNA/RNA sequencing data and chemotherapy response data for the Genomic of Drug Sensitivity in Cancer (GDSC) dataset were downloaded from the GDSC website (www.cancerrxgene.org). Processed DNA/RNA sequencing data and chemotherapy response data for the Cancer Cell Line Encyclopedia (CCLE) were downloaded from the CCLE website (portals.broadinstitute.org/ccle). The Cancer Dependency Map (DepMap) shares the same cell lines and therefore DNA/RNA sequencing data as CCLE, but independently evaluated chemotherapy response, which was obtained from the DepMap website (depmap.org). As recommended by DepMap, the MTS010 dataset was used for drug response data. In addition, we sought to identify published cohorts with pre-chemotherapy RNA profiling with pathologic response data. The majority of data we could find was in the setting of breast cancer neoadjuvant chemotherapy, where response could be assessed at the time of surgery. Our dataset evaluating clinical treatment response and the phenocopy signatures in breast cancer included 18 breast cancer cohorts with gene expression data downloaded from the Gene Expression Omnibus (GEO) with the following accession numbers: GSE22093, GSE25055, GSE41998, GSE20194, GSE4779, GSE8465, GSE66399, GSE16446, GSE20271, GSE18864, GSE25065, GSE32646, GSE164458, GSE22226, GSE192341, GSE163882, GSE34138, GSE32603, and one additional publicly available TNBC gene expression cohort^54^. Finally, we utilized gene expression and clinical response data from the I-SPY2 trial as an independent clinical validation cohort for our phenocopy signatures and breast cancer neoadjuvant therapy response, also downloaded from GEO (GSE194040).
