# Supplementary material for: A phenocopy signature of TP53 loss predicts response to chemotherapy
Source: NPJ Precis Oncol. 2024 Oct 2;8:220. doi: 10.1038/s41698-024-00722-7 (PMC11447220; doi:10.1038/s41698-024-00722-7)
Supplement: Supplementary file 1 — Supplemental Data [file 41698_2024_722_MOESM1_ESM.pdf]

SUPPLEMENTARY FIGURES

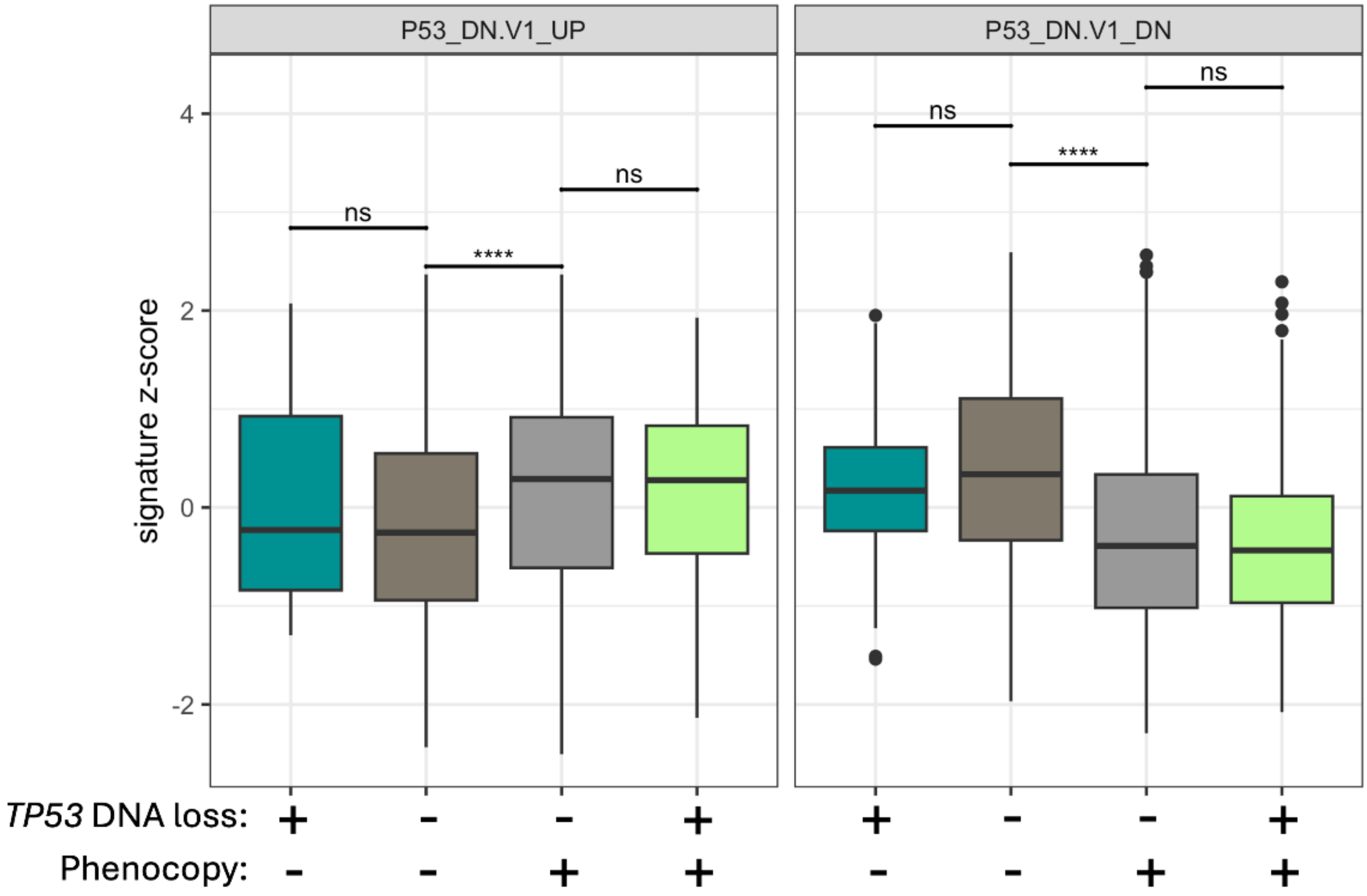

Supplementary Figure 1. GSVA single sample gene set analysis of the P53_DN signature from MSigDB (p53 mutated versus non-mutated cell lines in the NCI-60 collection) in TCGA samples.

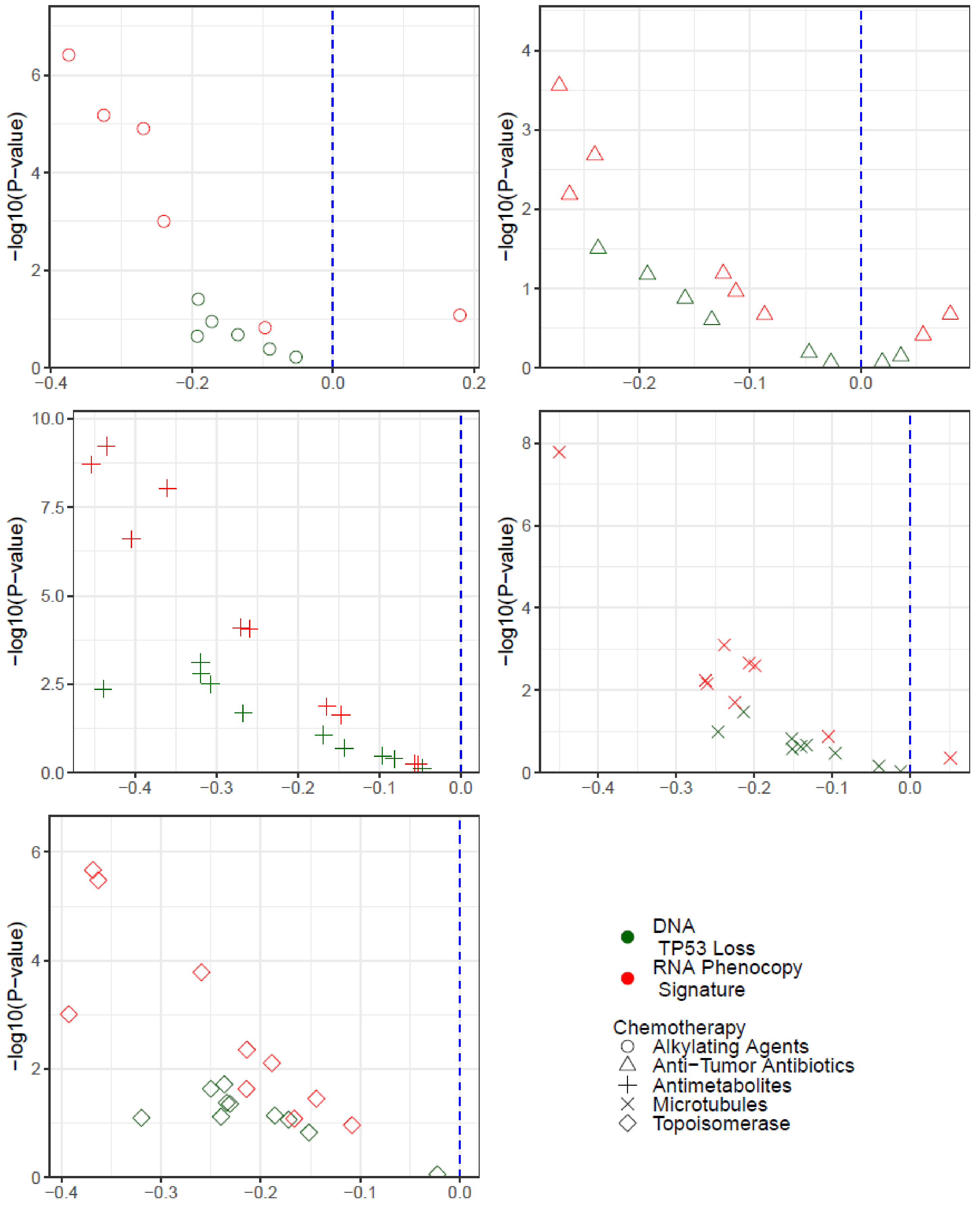

Supplementary Figure 2. *TP53*-loss phenocopy signature predicts chemotherapy response in vitro stratified by chemotherapy class. Linear models for cytotoxic chemotherapy response were used to assess

how much the RNA-based *TP53*-loss phenocopy signature added to DNA-based genomic *TP53* loss in the cell line datasets. Each model of a single chemotherapy is represented by two points, one for each independent variable (genomic *TP53* loss in green, *TP53*-loss phenocopy signature in red). The x-axis represents the linear coefficient, and the y-axis is the associated -Log10(p-value) of each independent variable in the linear model. Negative coefficients represent expected estimates, where the genomic or phenocopy *TP53*-loss status is associated with increased sensitivity to each chemotherapy. Data points in the upper-left quadrant therefore represent drugs for which the phenocopy signature most significantly contributed to predicting chemotherapy sensitivity.

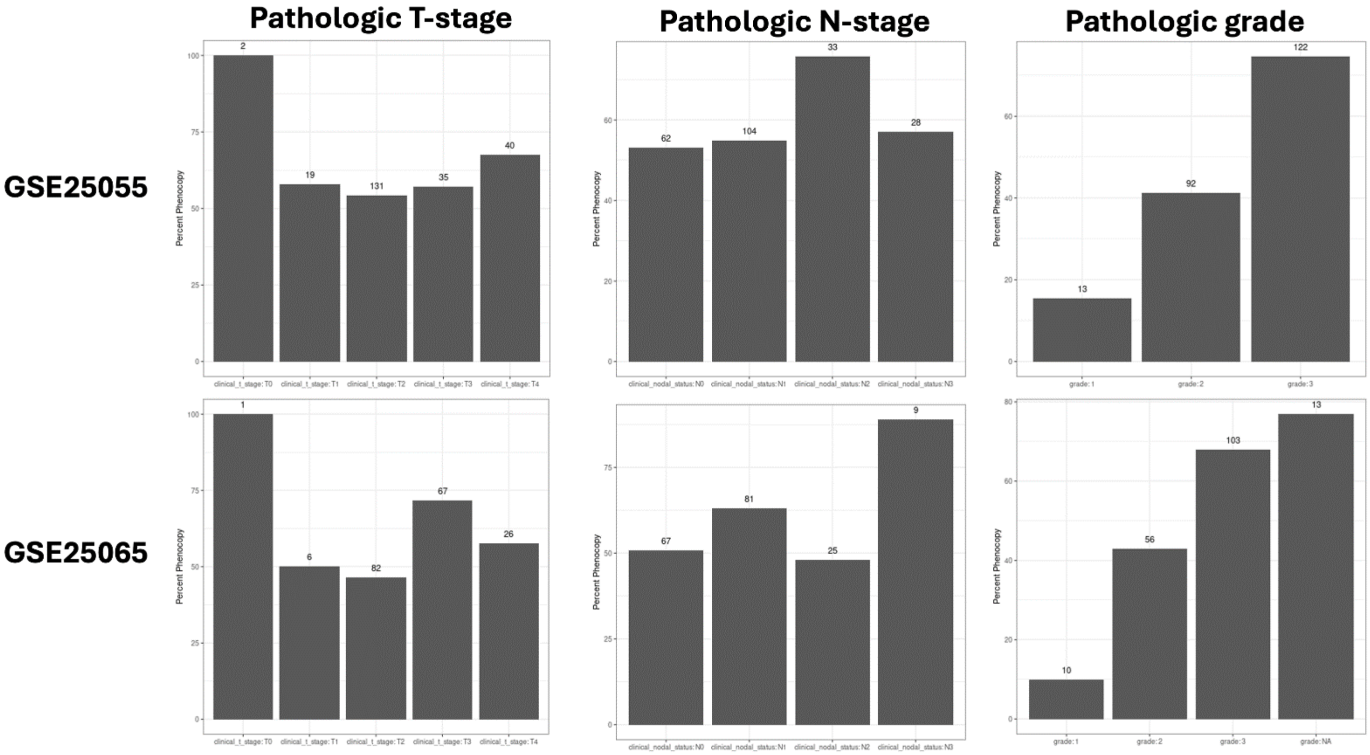

Supplementary Figure 3. Association of the *TP53* phenocopy signature and clinicopathologic variables

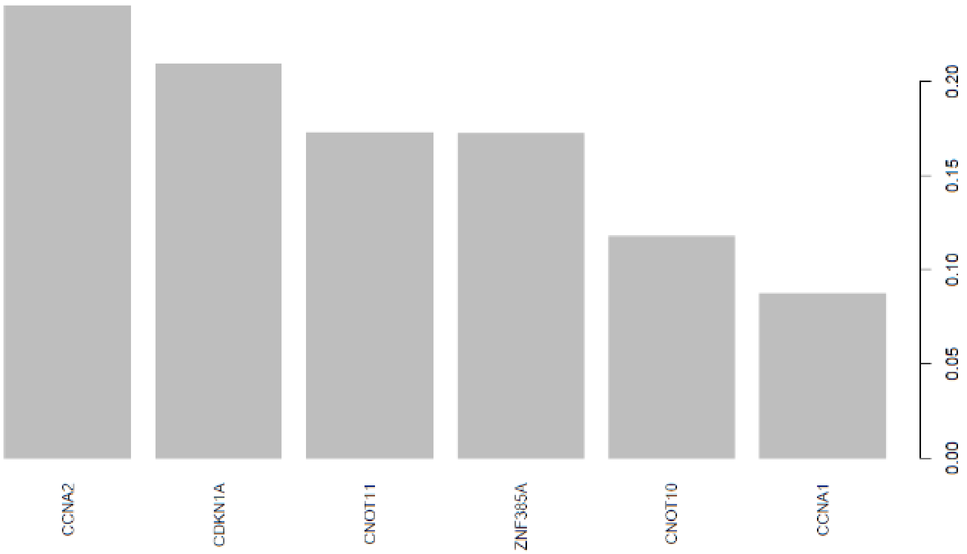

Supplementary Figure 4. Final set of genes in XGBoost and their relative importance in the model.

SUPPLEMENTARY TABLES

Accession	Year	Treatment	Data type	N - Total	N - Included	N - ER+	ER+ pCR%	N - ER-	ER- pCR%	HER2 status
GSE4779	2006	FEC	microarray	102	102	37	29.7	65	43.0	NR
GSE8465	2007	Gemcitabine, Doxorubicin, Cisplatin	microarray	46	36	16	31.2	19	36.8	26% HER2+
GSE16446	2009	Epirubicin	microarray	120	114	NA	NA	114	14.0	23.7% HER2+
GSE18864	2009	cisplatin	microarray	84	24	NA	NA	24	16.7	All HER2 normal
GSE22093	2010	FAC/or FEC	microarray	103	97	44	18.2	53	37.7	All HER2 normal
GSE25055	2010	FAC, FEC or AC followed by paclitaxel or docetaxel +-capecitabine	microarray	310	227[1]	131	6.1	96	36.5	All HER2 normal
GSE20194	2010	FAC+- paclitaxel	microarray	278	278	164	6.1	114	40.3	17% HER2+
GSE20271	2010	FAC+-paclitaxel	microarray	178	178	98	7.1	80	23.8	14.6% HER2+
GSE25065	2010	FAC, FEC or AC followed by paclitaxel or docetaxel +-capecitabine	microarray	198	182	113	16.8	69	33.3	All HER2 normal
GSE22226	2010	ISPY-1 platform	microarray	150	144	76	15.4	65	38.4	30% HER2+
GSE32603	2011	ISPY-1 platform	microarray	248	138[2]	85	12.9	52	46.1	30% HER2+
GSE32646	2011	FEC/paclitaxel	microarray	115	115	71	11.3	44	43.2	28.4% HER2+
GSE34138	2011	AC	microarray	178	177	120	9.2	57	47.3	All HER2 normal
GSE41998	2012	AC followed by taxane	microarray	279	270	103	12.6	167	16	9.8% HER2+
GSE66399	2015	FEC/paclitaxel with trastuzumab +- lapatinib	microarray	121	88	NA	NA	NA	NA	All HER2+; pCR% 30.6
GSE163882	2020	NR	RNAseq	222	222	105	22.9	117	47.9	28.4% HER2+
GSE164458	2021	AC followed by paclitaxel +- carboplatin +- veliparib	RNAseq	482	482	NA	NA	482	49.0	All HER2 normal
GSE192341	2021	AC, paclitaxel/ capecitabine, or both	RNAseq	87	85	35	8.6	50	42.0	All HER2 normal
VUMC	2023	Cisplatin and paclitaxel with everolimus +- placebo	RNAseq	45	44	NA	NA	45	44.4	All HER2 normal

Supplementary Table 1. Breast Cancer Neoadjuvant Chemotherapy Datasets
Samples overlapping with GSE22226 were excluded. 2. Pre-treatment (T1) samples only, and samples overlapping with GSE22226 excluded.
Abbreviations: 5-fluorouracil, adriamycin,cyclophosphamide (FAC); 5-fluorouracil, epirubicin, cyclophosphamide (FEC); adriamycin, cyclophosphamide (AC)

Variable	Coefficient	P-value
(Intercept)	-2.14	0.23
ER Status	-0.71	0.06
PR Status	-0.20	0.59
HER2 Status	0.86	0.57
Grade 2 vs. 1	0.04	0.97
Grade 3 vs. 1	1.48	0.17
T1 vs. T0	-0.35	0.81
T2 vs. T0	-0.47	0.72
T3 vs. T0	-0.46	0.73
T4 vs. T0	-1.54	0.27
N1 vs. N0	0.18	0.59
N2 vs. N0	-0.11	0.83
N3 vs. N0	0.50	0.32
Cohort (GSE25065 vs. GSE25055)	0.14	0.64
Phenocopy Status	0.76	0.02

Supplementary Table 2. Logistic regression predicting pCR in GSE25065 and GSE25055 in a multivariable logistic regression including the *TP53* phenocopy signature and clinicopathologic variables (pathologic grade, T/N-stage).
